# Clinical advances in epigenetic therapies for lymphoma

**DOI:** 10.1186/s13148-023-01452-6

**Published:** 2023-03-04

**Authors:** Allison C. Rosenthal, Javier L. Munoz, J. C. Villasboas

**Affiliations:** 1 Division of Hematology, Medical Oncology, Mayo Clinic, 5777 E. Mayo Blvd, Phoenix, AZ 85054 USA; 2 Mayo Clinic, 200 First St. SW, Rochester, MN 55905 USA

**Keywords:** B cell lymphoma, EZH2 inhibitor, Hodgkin lymphoma, T cell lymphoma

## Abstract

**Background:**

Advances in understanding of cancer biology, genomics, epigenomics, and immunology have resulted in development of several therapeutic options that expand cancer care beyond traditional chemotherapy or radiotherapy, including individualized treatment strategies, novel treatments based on monotherapies or combination therapy to reduce toxicities, and implementation of strategies for overcoming resistance to anticancer therapy.

**Results:**

This review covers the latest applications of epigenetic therapies for treatment of B cell, T cell, and Hodgkin lymphomas, highlighting key clinical trial results with monotherapies and combination therapies from the main classes of epigenetic therapies, including inhibitors of DNA methyltransferases, protein arginine methyltransferases, enhancer of zeste homolog 2, histone deacetylases, and the bromodomain and extraterminal domain.

**Conclusion:**

Epigenetic therapies are emerging as an attractive add-on to traditional chemotherapy and immunotherapy regimens. New classes of epigenetic therapies promise low toxicity and may work synergistically with other cancer treatments to overcome drug resistance mechanisms.

**Graphical Abstract:**

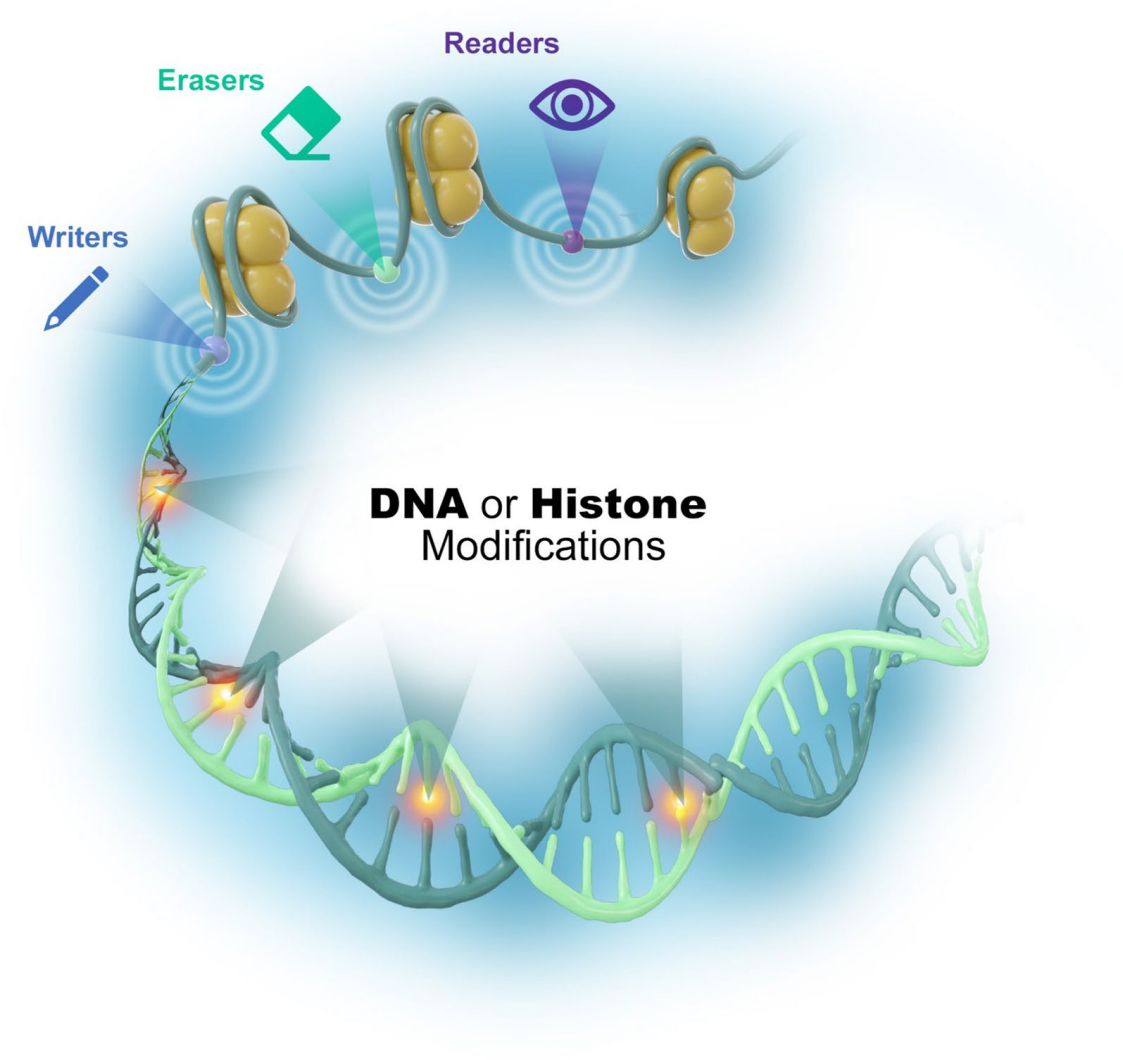

## Background

Non-Hodgkin lymphoma (NHL) is the seventh-most prevalent cancer and has the sixth-highest mortality rate among cancers in the USA [1]. Hodgkin lymphoma (HL) is the other main subtype of lymphoma. An estimated 8830 individuals in the USA will have been diagnosed with HL in 2021 [2]. Other subtypes of NHL include B cell, T cell, and natural killer cell lymphomas, with the most common B cell lymphoma subtypes being diffuse large B cell lymphoma (DLBCL; 31% of diagnosed lymphomas), follicular lymphoma (FL; 22%), and marginal zone lymphoma (8%) [1]. The prognoses for patients with HL and NHL are dependent on a variety of factors, such as age at diagnosis, cancer stage, lymphatic involvement, how well the patient performs normal daily activities (performance status), and levels of lactate dehydrogenase in the blood [3]. Although survival rates are dependent on these factors, the overall 5-year survival rate is 89% for patients with HL [2] and 73% for patients with NHL [2, 3]. Survival rates are globally increasing; however, the prognosis for people with relapsed lymphoma remains poor.

In patients with NHL or HL, common treatment options include chemotherapy, immunotherapy, and radiotherapy, used alone or in combination, and have high curative potential for many patients but come with significant challenges for a small subset of patients. Although chemotherapy is a well-established, effective method of treating lymphoma, many adverse events (AEs) are associated with chemotherapeutic agents due to their cytotoxic and nonselective mechanisms of action [4]. Some of the short-term impacts of chemotherapy, such as fatigue, nausea, hair loss, and loss of appetite, resolve quickly with lifestyle modifications and/or supportive therapy [5]. Supportive therapy or other approaches used to reduce the impact of chemotherapy-induced AEs are not effective in reducing the long-term impact of chemotherapy and may be associated with additional AEs. Some of the long-term impacts of chemotherapy that may or may not be controlled by supportive medicine include loss of fertility, secondary cancer development, and lung damage. In aggressive forms of lymphoma, such as DLBCL, most patients are older than 60 years when diagnosed and some are too frail for standard chemotherapies [6].

The anti-CD monoclonal antibody rituximab revolutionized the treatment and prognosis for CD20^+^ B cell malignancies and established immunotherapy as a valid treatment option for B cell malignancies [7]. Despite the success of rituximab, resistance to first-line rituximab in indolent B cell NHL and in relapsed or refractory (R/R) disease can occur [8]. Rituximab is indicated for treatment of patients with previously untreated or R/R B cell NHL [9]as monotherapy for patients with R/R FL, in combination with chemotherapy regimens containing cyclophosphamide, doxorubicin, vincristine, and prednisone (CHOP) for patients with untreated DLBCL, and with cyclophosphamide, vincristine, and prednisolone (CVP) for patients with untreated FL [9, 10]. For advanced-stage FL, common treatment regimens include rituximab alone or in combination with chemotherapy, such as CVP or CHOP [11], or a targeted therapy, such as rituximab plus lenalidomide (R^2^), as first-line therapy [12]. R^2^ is also used as a treatment option for multiple NHL subtypes [9, 10, 13]. Combination rituximab and bendamustine is also indicated for patients with indolent NHL [14]. Rituximab is a component in 58% of second-line FL combination therapies, and the majority of patients with R/R FL will receive rituximab again in subsequent lines of therapy [15]. For example, one study showed that 40% of patients with relapsed FL or low-grade NHL responded to re-treatment with rituximab after having received prior rituximab therapy [16]. However, rituximab rechallenge in patients with R/R NHL is associated with a shorter progression-free survival rate than patients naive to rituximab [10]. Therefore, alternatives to existing treatments are being sought, particularly with regard to targeted therapies [17]. More recently, glycoengineered type II anti-CD20 monoclonal antibodies, such as obinutuzumab, have shown superior response rates to rituximab, whether as monotherapy or in combination for R/R indolent lymphoma [18]. Unfortunately, this therapy is associated with increased side effects compared with rituximab monotherapy and may be better suited in combination with therapies with low risk of AEs [17, 18].

Subsequent advances in our understanding of the biology, genetics, and immunology of cancer led to development of targeted therapies and immunomodulatory drugs, including small molecule kinase inhibitors and monoclonal antibodies targeting proteins involved in cancer cell growth and mitogenic signaling. Although these therapies are more selective than chemotherapy and thus designed to generate fewer off-target effects [4], their use is associated with unique AEs related to their targets, such as cutaneous toxicity observed with epidermal growth factor receptor inhibitors [19], and hepatotoxicity, diarrhea, glucose regulation abnormalities (e.g., hyperglycemia) [20], as well as pneumonitis associated with phosphatidylinositol 3-kinase inhibitor use [21]. Emerging anticancer immunotherapeutic agents (e.g., immune checkpoint inhibitors, T cell therapies) are associated with immune-related AEs that can affect their safety and tolerability profiles [22, 23]. Issues of cytotoxicity, treatment resistance, and tolerability with the aforementioned therapies indicate additional need for new classes of therapeutics.

**Fig. 1:**
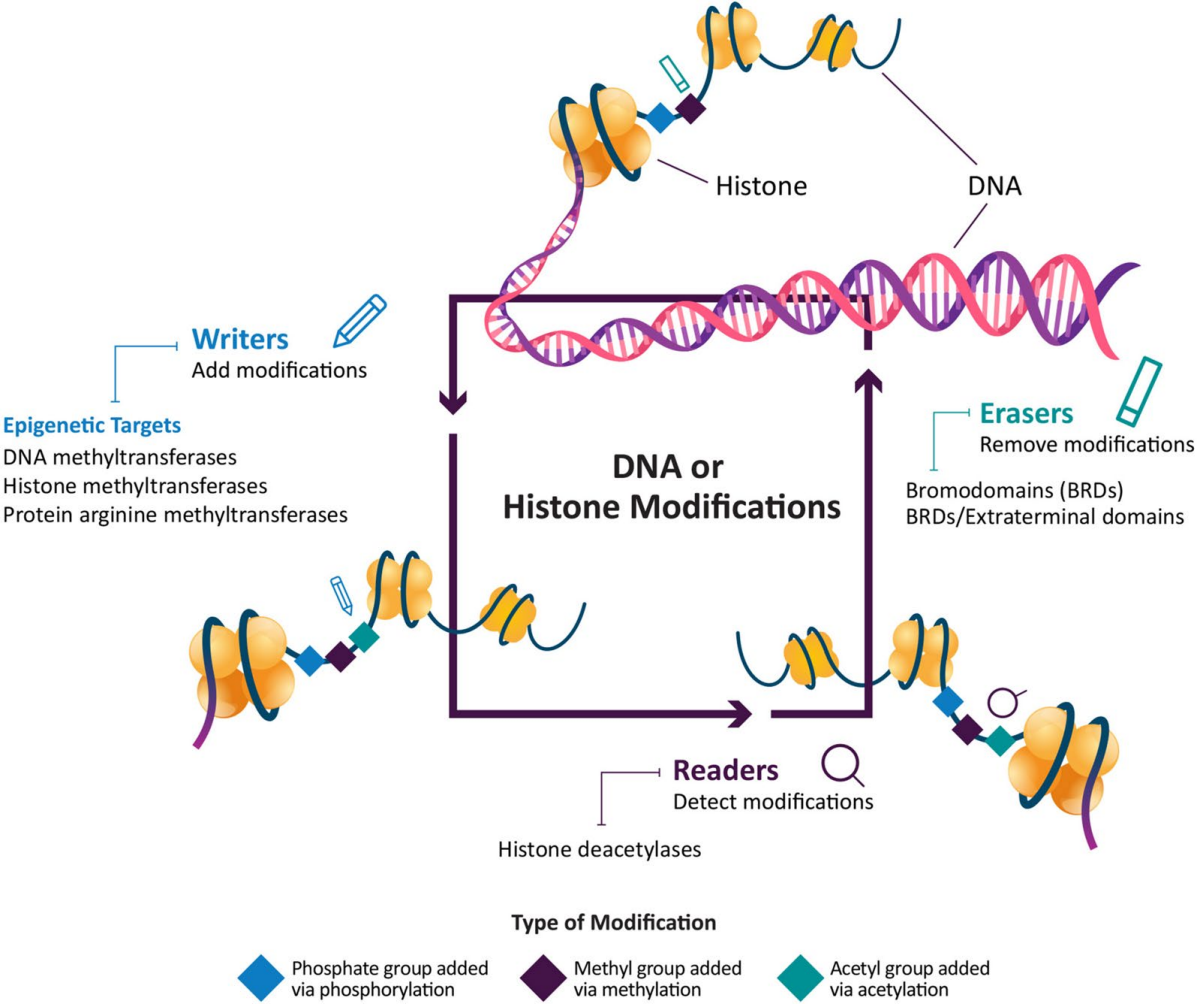
Overview of key epigenetic mechanisms and classes of epigenetic therapies

Epigenetic therapies are a selective way to treat cancer that avoid the cytotoxic AEs associated with chemotherapy and targeted therapies [24]. They are also a means of overcoming drug resistance pathways, making them an important complementary tool in the treatment of R/R lymphomas. Epigenetic modulators control gene expression and are involved in several cellular processes that depend on modification of nucleic acids and histones, including cellular growth and proliferation [25]. Epigenetic modifications include DNA methylation and histone acetylation/methylation, all of which regulate the accessibility of chromatin to transcription factors and other DNA-binding proteins (**Fig. ****1**) [25]. Many epigenetic processes are linked to oncogenesis and cancer proliferation [25]. Several epigenetic therapies are approved for treatment of lymphomas [26–30].

This review provides an in-depth summary of key clinical advances of epigenetic therapies for treatment of lymphoma in the last 5 years and the potentially synergistic benefits they provide when administered with other therapeutic agents. Classes of therapeutic agents covered include inhibitors of DNA N-methyltransferases (DNMTs), histone methyltransferases (HMTs), protein arginine methyltransferases (PRMTs), histone deacetylases (HDACs), bromodomain (BRD), and BRD/extraterminal domain (BET; Table 1) [31–43]. This review covers the safety profiles of key agents from these classes (Table 2).

**Table 1:** Summary of key epigenetic therapies by lymphoma type

Lymphoma type	Epigenetic inhibitor class	Key result	
		Monotherapy	Combination therapy
BCL	DMNT	No clinical benefit [31]	R/R DLBCL (azacitidine + vorinostat + gemcitabine/busulfan/melphalan): ORR 78% [32]
	EZH2	MT *EZH2* FL (tazemetostat): ORR 69% [33]	Data not yet available
	PMNT	Data not yet available	No studies
	HDAC	R/R DLBCL (fimepinostat): ORR 37% FL (vorinostat): ORR 47–49% [34, 35]	NHL (vorinostat + R-CVEP): ORR 41–57% [36, 37]
TCL	EZH2	TCL (valemetostat): ORR 80% [38]	No studies
	HDAC	CTCL (vorinostat): ORR 30% [39] CTCL (romidepsin): ORR 33–41% [100–102] PTCL (romidepsin): ORR 25% [40]	R/R PTCL (romidepsin + pralatrexate): ORR 71% [41]
HL	HDAC	Ineffective vs immunomodulating agents [42]	Vorinostat + mTOR inhibitor: ORR 33–55% [43]
	DMNT	No studies	Azacitidine + vorinostat + chemotherapy (gemcitabine/busulfan/melphalan): ORR 88% [32]

*BCL* B cell lymphoma; *CTCL* cutaneous T cell lymphoma; *DLBCL* diffuse large B cell lymphoma; *EZH2* enhancer of zeste homolog 2; *DMNT* DNA N-methyltransferase; *FL* follicular lymphoma; *HDAC* histone deacetylase; *HL* Hodgkin lymphoma; *mTOR* mechanistic target of rapamycin; *NHL* non-Hodgkin lymphoma; *ORR* objective response rate; *PMNT* phenylethanolamine N-methyltransferase; *PTCL* peripheral T cell lymphoma; *R-CVEP* rituximab, cyclophosphamide, vorinostat, etoposide, and prednisone; *R/R* relapsed or refractory; and *TCL* T cell lymphoma

**Table 2:** Summary of select grade ≥ 3 AEs by epigenetic therapy class

Epigenetic class	Agent	Indication	Grade ≥ 3 AE, incidence (%)
DNMT inhibitor	Azacitidine + vorinostat + chemotherapy	NHL, HL, TCL [32]	Mucositis (32), neutropenic fever (100), transient hyperbilirubinemia (18), transaminase elevation (25)
EZH2 inhibitor	Tazemetostat	FL [33]	Anemia (2), neutropenia (3), thrombocytopenia (3)
	Valemetostat	NHL, TCL [38]	Pneumonia (7), anemia,^a^ dysgeusia,^a^ decreased platelet count^a^
PRMT5 inhibitor	JNJ-64619178	NHL [73]	Anemia (17), neutropenia (6), thrombocytopenia (20)
HDAC inhibitor	Vorinostat	NHL [34, 35, 74]	Anemia (4–6), decreased platelet count (29), neutropenia (41), thrombocytopenia (6–48)
		CTCL [39, 75]	Anemia (1–8), fatigue (5), thrombocytopenia (5–19)
	Abexinostat	NHL, TCL [76]	Anemia (12), neutropenia (27), thrombocytopenia (80)
	Panobinostat	CTCL [77, 78]	Thrombocytopenia (17–20), neutropenia (9–20)
	Fimepinostat	DLBCL [79]	Neutropenia (16), thrombocytopenia (36)
	Romidepsin	CTCL, PTCL [40, 100–102]	Neutropenia (0–18), thrombocytopenia (0–23), lymphopenia (0–21), leukopenia (0–12)
BET inhibitor	Molibresib	NHL [80]	Thrombocytopenia (70)

*BET* bromodomain and extraterminal domain; *CTCL* cutaneous T cell lymphoma; *DLBCL* diffuse large B cell lymphoma; *DMNT* DNA N-methyltransferase; *EZH2* enhancer of zeste homolog 2; *FL* follicular lymphoma; *HDAC* histone deacetylase; *HL* Hodgkin lymphoma; *NHL* non-Hodgkin lymphoma; *PTCL* peripheral T cell lymphoma, *PRMT* protein arginine methyltransferase; and *TCL* T cell lymphoma

^a^Adverse events not listed by grade

## Epigenetic mechanisms targeted in cancer therapy

### Epigenetic writers

DNA hypermethylation in cancer cells is associated with silencing of tumor suppressor genes and activation of oncogenes, and it is one of the most characterized epigenetic mechanisms in cancer [44]. Targeting epigenetic writers, such as DNMTs, PRMTs, and HMTs, are an attractive strategy to restore the epigenomic regulation of cellular proliferation and halt cancer cell growth (Fig. 1) [25]. DNA methyltransferases transfer a methyl group from S-adenosylmethionine to the cytosine ring in a cytosine–phosphate–guanine dinucleotide pair in the C5 position to create 5-methylcytosine, a molecule that regulates gene transcription [44]. Studies show that DNMT inhibitors induce hypomethylation and upregulation of genes involved in DNA transcription, RNA processing, and ribosomal function as their primary therapeutic effect, although it is unclear if the modest increase in the rate of hypomethylation caused by an inhibitor is enough to affect cellular functions [45, 46]. *DNMT* mutations are common in many kinds of cancer cells, including lymphomas, and studies have shown that a higher baseline level of methylation exists in cancerous cells compared with noncancerous cells, even when cells stop dividing [47, 48].

Histone methyltransferases play a similar role to that of DNMTs in cancer cell biology, catalyzing the transfer of 1 or more methyl groups to lysine and arginine residues on histone proteins [49]. Depending on the site of methylation, histones can promote or repress gene transcription [50]. The HMT EZH2 has emerged as an attractive therapeutic strategy for various malignancies because of its noncytotoxic mechanism of action and hypothesized role in overcoming mechanisms of chemoresistance [51–53]. Enhancer of zeste homolog 2 is the enzymatic subunit of polycomb repressive complex 2 that catalyzes 1 to 3 methylations of Lys27 on histone H3, which serves to repress gene transcription [54, 55]. The gain-of-function EZH2 mutation at tyrosine 641 within the catalytic SET domain increases H3K27 trimethylation and decreases H3K27 monomethylation [54, 56]. However, wild-type germinal center-derived lymphomas also retain dependency on EZH2 to proliferate and repress plasma cell differentiation [57]. EZH2 plays a role in many processes within the tumor microenvironment, including CD4^+^, natural killer, and CD8 T cell differentiations and functions [58].

Other potential epigenetic writer targets are protein arginine methyltransferase 5 (PRMT5) and lysine methyltransferase 2 (KMT2). PRMT5 is a subtype of PRMT that catalyzes the methylation of mono- and symmetric dimethylarginine, which can repress or promote gene transcription. PRMT5 is one of the most overexpressed PRMT types in cancer cells, regulating B cell proliferation and survival, and germinal cell formation and expansion, hence its emergence as a possible therapeutic target [59, 60]. KMT2, a lysine methyltransferase, is considered to be the main lysine 4 of the core histone H3 (H3K4) methyltransferase [61]. KMT2 positively regulates gene expression and mutations, in particular subsets of the KMT2 family, and has been implicated in hematologic malignancies owing to induction of H3K4me3 to the promoter of genes associated with hematopoietic cell development and differentiation [62].

### Epigenetic erasers

Epigenetic erasers are proteins that remove DNA and histone protein modifications produced by epigenetic writers to regulate gene expression (Fig. 1) [25]. Histone and nonhistone protein acetylation is regulated through opposing functions of histone acetyltransferases and HDACs [25, 42, 63]. Histone acetylation promotes cancer cell growth by increasing the rate of gene expression of oncogenes, such as B cell lymphoma 6 (BCL6), which regulates oncogenes implicated in FL, DLBCL, and B cell lymphoproliferative disorders [25, 42, 63, 64]. Histone deacetylases are made up of 4 classes that are all potential anticancer targets, although some are zinc dependent (classes 1, 2a/b, and 4) or nicotinamide–adenine–dinucleotide dependent (class 3) [42]. Studies show HDAC inhibitors reactivate tumor suppressor genes and promote an immune response [65].

Another epigenetic eraser, histone demethylases (KDM), removes the arginine and lysine residues added by HMTs [66]. A particular subfamily of KDMs, KDM5, can remove tri- and dimethyl marks from H3K4. KDM5 can either activate or suppress transcription depending on the methylation site and has been shown to be involved in the regulation of oncogenes [66].

### Epigenetic readers

Epigenetic readers are composed of domains that recognize and bind to specific post-translational DNA or histone modifications. These modifications include BRD-containing proteins that recognize acetylated histone residues, methyl cytosine–phosphate–guanine-binding domains that recognize methylated cytosine–phosphate–guanine, and chromodomains that recognize methylated lysine (Fig. 1) [25]. The BET family of epigenetic readers plays a critical role in cancer development by activating and potentiating expression of key oncogenes [25, 63]. The BET family comprises 4 members, including BRD-containing proteins 2 (BRD2), 3 (BRD3), 4 (BRD4), and t (BRDt) [25], although targeting BRD4 is thought to be the primary cause of the anti-oncogenic effects of BET inhibitors [67].

## Epigenetic therapies for lymphomas

### B cell non-Hodgkin lymphomas

#### DNA methyltransferase inhibitors

Two DNMT inhibitors, azacitidine and decitabine, are approved by the US Food and Drug Administration (FDA) and the European Medicines Agency for treatment of acute myeloid leukemia and myelodysplastic syndrome [27, 68, 69], but studies thus far have shown no evidence that decitabine promoted hypomethylation in individuals with DLBCL [31].

Although decitabine is ineffective as a monotherapy [31], a phase 1/2 study combined decitabine with chemotherapy in 6 patients with DLBCL, NHL, and HL who previously experienced progressive disease (PD) on chemotherapy. Two participants maintained stable disease (SD), and the remainder experienced PD after several months [70]. In phase 1 studies, DNMT inhibitors CC-486 (oral azacitidine) and azacitidine rendered chemotherapy-resistant DLBCLs sensitive to CHOP [71, 72]. In 26 participants with R/R DLBCL eligible for high-dose therapy, azacitidine followed by vorinostat in combination with gemcitabine, busulfan, and melphalan as second-line therapy yielded an objective response rate (ORR) of 78% and a complete response (CR) rate of 55% [32]. Common AEs reported with this regimen include mucositis, dermatitis, and transient hyperbilirubinemia, which resolved after 1 week on treatment [32–35, 38, 39, 73–80].

#### Enhancer of zeste homolog 2 inhibitors

In early clinical trials, tazemetostat, an EZH2 inhibitor that also reduces methyltransferase activity in *EZH2* mutant and wild-type FL [81], produced CR rates of 38% in participants with NHL [82] and 33% in participants with R/R DLBCL [83]. In a phase 2 trial in 99 participants with R/R FL, those with wild-type *EZH2* FL (*n* = 54) had an ORR of 35% and participants with mutant *EZH2* FL (*n* = 45) had an ORR of 69%. Durations of response in wild-type *EZH2* FL were 13.0 months and 10.9 months in the mutant *EZH2* FL group [33]. In 2020, tazemetostat received accelerated regulatory approval from the FDA for treatment of R/R FL. Grade ≥ 3 AEs associated with tazemetostat were low and included thrombocytopenia (3%), neutropenia (3%), and anemia (2%) [33]. By contrast, grade 3–4 AE rates of  > 30% for thrombocytopenia were reported with other classes of epigenetic agents [79, 80, 84]. Following its approval as monotherapy, tazemetostat is undergoing evaluation in combination with rituximab (NCT04762160), in combination with lenalidomide and rituximab (NCT04224493) for treatment of R/R FL, and in combination with prednisolone for treatment of R/R DLBCL (NCT01897571).

Another EZH2 inhibitor with reported clinical data is valemetostat, an oral dual inhibitor of EZH2 and enhancer of zeste homolog 1 (another methyltransferase) [85]. An ORR of 53% was achieved in the first-in-human clinical trial of 15 participants with B or T cell lymphoma [38, 63]. Several other EZH2 inhibitors are in ongoing clinical studies for treatment of NHL and include CPI-0209 (NCT04104776), HH2853 (NCT04390737), and PF-06821497 (NCT03460977; Table 3) [86, 87].

**Table 3:** Ongoing clinical trials of EZH2 inhibitors

Clinical trial	Therapeutic agent	Key study population
SYMPHONY-1 Phase 1b/3 NCT04224493 [86]	Tazemetostat + lenalidomide + rituximab	R/R FL
SYMPHONY-2 Phase 2 NCT04762160 [87]	Tazemetostat + rituximab	R/R FL
Phase 1/2 NCT01897571	Tazemetostat + prednisolone	Advanced solid tumors (single-agent tazemetostat) R/R DLBCL (combination therapy)
Phase 2 NCT04842877	Valemetostat	R/R aggressive B cell lymphomas, transformed indolent lymphoma, FL, MCL, MZL, HL
Phase 1/2 NCT04104776	CPI-0209	Advanced solid tumors R/R DLBCL
Phase 1 NCT04390737	HH2853	Advanced solid tumors R/R DLBCL, FL
Phase 1 NCT03460977	PF-06821497	R/R SCLC (combination therapy with SOC), CRPC (combination therapy with SOC), FL (single agent), DLBCL (single agent)

*CRPC* castration-resistant prostate cancer; *DLBCL* diffuse large B cell lymphoma; *FL* follicular lymphoma; *HL* Hodgkin lymphoma; *MCL* mantle cell lymphoma; *MZL* marginal zone lymphoma; R/R relapsed or refractory; *SCLC* small cell lung cancer; and *SOC* standard of care

#### Protein arginine methyltransferase inhibitors

Three PRMT5 inhibitors—GSK3326595, JNJ-64619178, and PRT811—are in clinical development. GSK3326595 is being evaluated in a dose-escalation study of patients with solid tumors and NHL (NCT02783300). JNJ-64619178 is being studied in an ongoing phase 1 trial (NCT03573310) in which it induced partial responses and SD in participants with NHL and solid tumors [73]. PRT811 is being studied in a phase 1 trial for treatment of approximately 75 participants with either central nervous system lymphomas, advanced solid tumors, or recurrent high-grade gliomas (NCT04089449). Of the 54 patients enrolled in the JNJ-64619178 trial, preliminary clinical results indicate that 20% experienced grade ≥ 3 thrombocytopenia, 17% experienced grade ≥ 3 anemia, and 6% experienced grade ≥ 3 neutropenia [73].

#### Histone deacetylase inhibitors

First-generation HDAC inhibitors such as vorinostat are nonselective toward the 4 classes of HDAC enzymes, but selective HDAC inhibitors are being developed as a promising treatment class that may result in fewer off-target effects than pan-HDAC inhibitors [88].

Monotherapy with HDAC inhibitors has demonstrated modest clinical activity in several studies [34, 35, 74, 76, 79]. Vorinostat, an inhibitor of class 1/2 HDAC enzymes, was tested as a monotherapy in a phase 2 study of patients with relapsed DLBCL, but 16 of 18 enrolled patients experienced PD [74]. By contrast, vorinostat monotherapy produced an ORR of 47% to 49% in participants with FL and an ORR of 22% in participants with marginal zone lymphoma (*n* = 9) [34, 35]. None of the participants with mantle cell lymphoma in either study (*n* = 4 and *n* = 9) achieved a clinical response with vorinostat monotherapy. The most common grade 3/4 AE associated with vorinostat was thrombocytopenia [34, 35, 74]. Another HDAC inhibitor, mocetinostat, was tested in a phase 2 study of patients with R/R DLBCL and FL, but the drug failed to reach the threshold for clinical efficacy (> 20% ORR) [89]. Abexinostat, a pan-HDAC inhibitor, was clinically active as monotherapy in a phase 2 study of patients with FL (ORR, 56%) or DLBCL (ORR, 31%) [76]. Similar to vorinostat, the most common grade 3/4 AEs were thrombocytopenia (80%) and neutropenia (27%) [76].

Fimepinostat, a first-in-class, dual-target inhibitor of phosphatidylinositol 3-kinase (PI3K) class I and pan-HDAC enzymes, achieved an ORR of 37% when administered as monotherapy in 25 participants with R/R DLBCL [79]. Of these participants, 36% experienced grade ≥ 3 thrombocytopenia and 16% experienced grade ≥ 3 neutropenia, resulting in a safety profile comparable with other HDAC inhibitors [79]. The FDA granted fimepinostat fast-track status in 2018 for treatment of R/R DLBCL [29].

Most HDAC inhibitors have low responses in B cell lymphomas when used as monotherapy, but they demonstrate synergy with other drugs; however, this synergy is tempered by poor safety profiles. Vorinostat in combination with rituximab or rituximab plus cyclophosphamide, etoposide, and prednisone achieved an ORR of 41% to 57% in participants with R/R B cell NHL, including those with DLBCL. The most frequent grade ≥ 3 AEs included lymphopenia (25–90%), fatigue (14–32%), and lowered platelet (18–41%) and neutrophil (11–52%) counts [36, 37]. In another phase 1/2 study, vorinostat in combination with rituximab plus CHOP (R-CHOP) achieved an ORR of 81% in participants with R/R DLBCL (*n* = 63), but participants assigned to the study combination had a high incidence of grade ≥ 3 neutropenia (63%), thrombocytopenia (36%), and sepsis (19%) that led investigators to recommend against general clinical use of vorinostat with R-CHOP [90]. Vorinostat in combination with rituximab, ifosfamide, carboplatin, and etoposide for treatment of R/R NHL demonstrated efficacy in DLBCL (ORR, 67%; *n*/*N* = 4/6), mantle cell lymphoma (ORR, 60%; *n*/*N* = 3/5), and FL (ORR, 100%; *n*/*N* = 3/3). The most common grade ≥ 3 AEs at the maximum tolerated dose included hypophosphatemia (27%), febrile neutropenia (27%), and infection (27%) [91]. Abexinostat is being studied in combination with ibrutinib in a phase 1 trial of patients with DLBCL or mantle cell lymphoma (NCT03939182). Key AEs associated with HDAC inhibitors in patients with B cell lymphomas include grade ≥ 3 fatigue, neutropenia, and thrombocytopenia (which can affect ≥ 30% of patients), and gastrointestinal issues (e.g., diarrhea, dehydration, anorexia) [88, 92].

#### Bromodomain and extraterminal domain inhibitors

Bromodomain and extraterminal domain inhibitors that target BRD2, BRD3, and BRD4 have been studied in the clinical setting. A phase 1 study of birabresib, a BRD2/BRD3/BRD4 inhibitor, reported responses in 3 of 22 patients with DLBCL whose disease progressed on other treatments. However, because of toxicities (e.g., thrombocytopenia) associated with the chosen study drug dose, a lower dose was selected for additional studies [84].

Responses with other BET inhibitors were also disappointing. In a phase 1/2 study, INCB057643 generated an ORR of 33% in patients with R/R FL (*n*/*N* = 1/3) [93]. BAY 1238097 was ineffective against refractory malignancies of any type, and its trial was halted due to toxicity issues and lack of efficacy [94]. Interim results from a phase 1 study of molibresib, another BET inhibitor tested in patients with NHL [63, 80], found that 70% of participants (*n*/*N* = 19/27) experienced grade ≥ 3 thrombocytopenia [80]. In addition to thrombocytopenia, gastrointestinal toxicity and diarrhea are commonly reported nonhematologic AEs in patients receiving BET inhibitors [80, 84, 95–97].

Interim results from a phase 1 study of 44 participants showed that treatment with CPI-0610 achieved CRs in 3 of 24 participants with R/R DLBCL and 1 partial response in 8 participants with R/R FL. Eleven participants with R/R lymphomas showed ongoing partial responses (*n* = 5) or SD (*n* = 6) [97]. One participant developed grade 4 thrombocytopenia, and another developed grade 3 diarrhea.

### Hodgkin lymphoma

#### DNA methyltransferase inhibitors

A small clinical study that included 8 patients with HL examined the efficacy of azacitidine followed by vorinostat with chemotherapy (gemcitabine, busulfan, and melphalan) as second-line therapy [32]. This combination yielded CRs in 7 of 8 participants with HL [32]. Furthermore, event-free and overall survival rates were high (76% and 95%, respectively) after a median follow-up period of 15 months. However, all participants developed neutropenic fever [32]. In addition, an ongoing phase 1 study is evaluating azacitidine followed by tumor assisted antigen-specific cytotoxic T cell lymphocytes in patients with HL and NHL (NCT01333046).

#### Histone deacetylase inhibitors

Several HDAC inhibitors have been tested as monotherapies in patients with R/R HL in clinical trials, including panobinostat [98], vorinostat (NCT00132028), givinostat (NCT00496431), resminostat (NCT01037478), mocetinostat (NCT00358982 [trial terminated]), abexinostat (NCT00724984, NCT01149668), ricolinostat (NCT02091063), entinostat (NCT00866333), and tinostamustine (NCT02576496). Panobinostat produced an ORR of 27% in 129 participants with R/R HL [98]. However, in the other completed or terminated clinical trials, HDAC inhibitors generated relatively low ORRs and comparable progression-free survival versus other targeted therapies or immunomodulatory antibodies that target immune checkpoint pathways such as programmed cell death 1 (PD-1) or its ligand (PD-L1) [42].

Vorinostat is also being evaluated as part of a combination therapy regimen in patients with R/R HL. When combined with the mechanistic target of rapamycin inhibitors everolimus or sirolimus, vorinostat produced moderate ORRs (55% and 33%, respectively) in a dose-escalation study of patients with R/R HL [43]. Key AEs included grade ≥ 3 thrombocytopenia in 67% of participants treated with vorinostat plus everolimus and in 55% of participants treated with vorinostat plus sirolimus [43]. Vorinostat in combination with rituximab plus ifosfamide, carboplatin, and etoposide also demonstrated effectiveness for treatment of patients with R/R HL (ORR, 88%; *n*/N = 7/8) [91].

### T cell lymphomas

#### DNA methyltransferase inhibitors

The study described earlier [32] with azacitidine followed by vorinostat with chemotherapy as second-line therapy also included 8 patients with R/R peripheral T cell lymphoma (PTCL; *n* = 3; anaplastic large-cell lymphoma; *n* = 2 natural killer T cell lymphoma, *n* = 2; angioimmunoblastic T cell lymphoma, *n* = 1). The regimen yielded a CR rate of 100% in participants with T cell lymphoma and measurable disease (*n*/*N* = 2/2) [32]. A small retrospective series of 12 patients with angioimmunoblastic T cell lymphoma, 11 of whom had R/R disease and 1 who was treatment naive, were treated with azacitidine and experienced a 75% ORR, median progression-free survival of 15 months, and a median overall survival rate of 21 months [99]. Several studies looking at DMNT inhibitors in combination with other treatments are planned or recruiting patients.

#### Enhancer of zeste homolog 2 inhibitors

Valemetostat achieved an ORR of 80% in early clinical trials among 15 patients with T and B cell lymphoma; however, the study examined 2 patients with adult T cell lymphoma, 2 with angioimmunoblastic T cell lymphoma, and 1 with PTCL not otherwise specified [38]. Key AEs included decreased platelet count (73%), anemia (47%), decreased lymphocyte count (40%), dysgeusia (47%), and diarrhea (27%) [38].

#### Histone deacetylase inhibitors

Several nonselective HDAC inhibitors have been studied as monotherapies for treatment of T cell lymphomas. Abexinostat produced an ORR of 40% in a phase 2 study of 15 participants with T cell lymphomas [76]. Oral vorinostat yielded an ORR of 24% to 30% in phase 2 studies in participants with refractory and progressive cutaneous T cell lymphoma (CTCL) and was approved for treatment of CTCL by the FDA in 2006 [39, 75]. Panobinostat, a pan-HDAC inhibitor, produced an ORR of 67% in participants with advanced CTCL (*n*/N = 6/10) when administered as oral monotherapy [78]; however, in a larger phase 2 cohort of 139 participants with refractory CTCL, the ORR was 17% [77]. Romidepsin, an intravenously administered selective HDAC class 1 inhibitor, produced an ORR of 33% to 41% in patients with CTCL [100–102]. In patients with CTCL, the most common grade 3/4 toxicities associated with panobinostat with or without abexinostat were thrombocytopenia (20–80%) and neutropenia (9–27%) [76–78]. However, grade 3/4 AE rates were lower with vorinostat: thrombocytopenia (5–19%), anemia (1–8%), and fatigue (5%) [39, 75].

Histone deacetylase inhibitors have also been tested as monotherapies for treatment of R/R PTCL, although the reported response rates were modest. Belinostat, a pan-HDAC inhibitor, demonstrated an ORR of 26% in participants with R/R PTCL (*n*/*N* = 31/120) [103] and an ORR of 46% in participants with R/R angioimmunoblastic T cell lymphoma (*n*/*N* = 10/22) [104]. Romidepsin produced an ORR of 25% in patients with R/R PTCL (*n* = 130) [40, 105]. After demonstrating clinical efficacy in phase 2 studies [102, 106], romidepsin was approved for treatment of R/R CTCL by the FDA in 2009 and for R/R PTCL in 2011, although the company withdrew the PTCL indication in 2021 owing to lack of efficacy in subsequent phase 3 trials [27, 28, 107]. Chidamide, a selective HDAC class 1 inhibitor, achieved an ORR of 39% when administered as oral monotherapy in 256 participants with R/R PTCL [108]. As with the other HDAC inhibitors discussed, hematologic events were the most common grade 3/4 AEs, with event rates generally below 30% [40, 102, 103, 106, 108].

As with other classes of epigenetic agents, HDAC inhibitors demonstrate slightly higher clinical activity in patients with T cell lymphomas in combination than when administered as monotherapies. As combination therapy with CHOP, vorinostat achieved an ORR of 100% in 12 participants with untreated PTCL [109]. Chidamide demonstrated an ORR of 51% when combined with chemotherapy in 127 participants with R/R PTCL [108]. Panobinostat in combination with bortezomib produced an ORR of 43% in participants with R/R PTCL (*n*/*N* = 10/23) [110]. Romidepsin combined with the antimetabolite pralatrexate demonstrated an ORR of 71% in a phase 1 study in participants with R/R PTCL [41]. Romidepsin in combination with oral azacitidine achieved an ORR of 61% in participants with R/R PTCL (*n*/*N* = 15/25) [111]. Key grade ≥ 3 AEs associated with HDAC inhibitors when used as combination therapy include thrombocytopenia (48%), neutropenia (40%), lymphopenia (32%), and anemia (16%) [111].

## Future directions

Although several classes of epigenetic treatments have shown moderate antitumor activity as monotherapies, combination regimens with chemotherapy generally had better clinical activity than monotherapy (Table 2). The success of PRMT5 inhibitors (GSK3326595 [112, 113], JNJ-64619178 [114], and PRT811 [115]) and KDM5 inhibitors [116] in preclinical and early phase studies may lead to the development of new classes of selective epigenetic therapies. Possible future combination therapies could include the EZH2 inhibitor tazemetostat and chemotherapies or antibodies. Combination of the HDAC inhibitor vorinostat with phosphatidylinositol 3-kinase inhibitors has demonstrated antitumor effects in preclinical studies with NHL cells [117]. DNA methyltransferase inhibitors have been shown to resensitize CHOP-resistant DLBCL to chemotherapy, and other epigenetic therapies may exploit similar mechanisms [71, 72]; for example, BET inhibitors have shown modest efficacy as monotherapy for treatment of B cell lymphomas [93, 97], but future studies of these inhibitors in combination with chemotherapy may allow higher rates of efficacy at lower doses to reduce the severity of clinical effects.

Studies are also beginning to show that epigenetic modulators can impact lymphoma therapy through promotion of antitumor immunity [118]. In the future, epigenetic therapy is likely to be part of a combination approach using multiple classes of treatment to simultaneously overcome drug resistance mechanisms and boost the patient’s immune system.

Many epigenetic therapies could be more successful in the clinical setting if they were individualized to patient biomarkers. An example of this is the difference in response rates between patients with wild-type and mutant *EZH2* FL treated with the EZH2 inhibitor tazemetostat (ORRs of 35% and 69%, respectively [33]), findings that suggest additional research to uncover epigenetic biomarkers is needed to identify patients more likely to benefit from these agents. As such, investigators should consider including epigenetic biomarker end points in their clinical trial designs.

## Conclusions

Older classes of epigenetic therapies, such as pan-HDAC inhibitors, are associated with high rates of toxicity and poor target selectivity, but newer classes, such as EZH2 inhibitors, have higher selectivity and better safety profiles and are being tested in a variety of lymphomas. By offering potential synergies with chemotherapies, kinase inhibitors, and antibodies, epigenetic agents can overcome chemoresistance and improve outcomes for patients with lymphomas, while their strong target selectivity can reduce the AEs associated with early epigenetic therapies.

Understanding the distinct mechanisms of emerging epigenetic therapies and the patient subpopulations they are most likely to benefit is key to their clinical implementation for treatment of lymphomas. The diversity of epigenetic therapies allows tailoring treatments to an individual patient’s tumor characteristics and needs and has the potential to greatly improve patient survival and quality of life.

## Data Availability

Not applicable.
